# Supplementary material for: Network based meta-analysis prediction of microenvironmental relays involved in stemness of human embryonic stem cells
Source: PeerJ. 2014 Oct 23;2:e618. doi: 10.7717/peerj.618 (PMC4217173; doi:10.7717/peerj.618)
Supplement: Table S2 — ALL R is the randomised network from the ALL interactome while C+S R is the randomised network from the C+S interactome (C: common part; S: specific part; R: random). Power law of the degree distribution: with R2, the polynomial regression coefficient (degree 2). [file peerj-02-618-s003.docx]

|  | **ALL** | **ALL R** | **S+C** | **S+C R** |
| --- | --- | --- | --- | --- |
| **Number of nodes N** | 702 | 702 | 209 | 209 |
| **Number of edges L** | 3201 | 3201 | 371 | 371 |
| **Connected components** | 13 | 3.4 | 16 | 6.4 |
| **Average degree <k>** | 9.12 | 9.12 | 3.55 | 3.55 |
| **Clustering coefficient <C>** | 0.37 | 0.06 | 0.27 | 0.05 |
| **Characteristic path length <l>** | 3.67 | 3.15 | 4.17 | 3.81 |
| **α** | 292.1 | / | 110.7 | / |
| **γ** | -1.3 | / | -1.5 | / |
| **R2** | 0.841 | / | 0.914 | / |
